# Acquired Hemophilia A After Multiple Transfusions Following Trauma

**DOI:** 10.7759/cureus.50295

**Published:** 2023-12-10

**Authors:** Krisha G Hidalgo, Danielle Z Azani, Robert Fincher, Andrew McCague

**Affiliations:** 1 Clinical Sciences, College of Osteopathic Medicine of the Pacific, Western University of Health Sciences, Pomona, USA; 2 Surgery, Desert Regional Medical Center, Palm Springs, USA; 3 Trauma, Desert Regional Medical Center, Palm Springs, USA; 4 Trauma and Acute Care Surgery, Desert Regional Medical Center, Palm Springs, USA

**Keywords:** acquired hemophilia a, factor viii, auto immune, feiba, factor viii inhibitors, acquired coagulation disorders

## Abstract

Acquired hemophilia A (AHA) is a coagulative disorder that is caused by the presence of inhibitors of factor VIII (FVIII). The presence of coagulation factor inhibitors can lead to severe episodes of bleeding in patients with no previous history of bleeding conditions. We present the clinical case of a man with severe bleeding two weeks after falling from a bicycle. The patient denied any previous history of bleeding disorders. The case clinically presented with a large retroperitoneal hematoma and continued to show signs of active bleeding even after multiple transfusions were administered. Coagulation studies showed an elevated inhibitor titer of 24.4 BU/mL (normal range is below 5 BU/mL) and a reduced FVIII activity level of 2% (normal range is between 50% to 150%), providing evidence of AHA. Hemostatic and immunosuppressive agents were then administered to the patient, whose condition improved in response to the treatments.

## Introduction

Acquired hemophilia A (AHA) is a hemorrhagic condition that is characterized by the presence of inhibitors of factor VIII (FVIII). These inhibitors can lead to severe and uncontrolled bleeding in patients who often have no prior history of bleeding disorders [1]. FVIII inhibitors are polyclonal immunoglobulins that act either by increasing clearance of the factor or by inhibiting the factor’s clotting activity, providing a regulatory mechanism to control the coagulation cascade. AHA is a rare condition with an annual incidence of approximately 1.5 in one million individuals [2]. This disorder commonly affects elderly patients above the age of 65 years old, and often occurs in the setting of trauma, malignancy, autoimmune conditions, or seen in younger populations during pregnancy [1-5]. There is a high mortality and morbidity risk in this condition, due to a delay in diagnosis and sequelae of the condition which can lead to bleeding that cannot be controlled or in vital organs such as the brain [6].

## Case presentation

**Figure 1:**
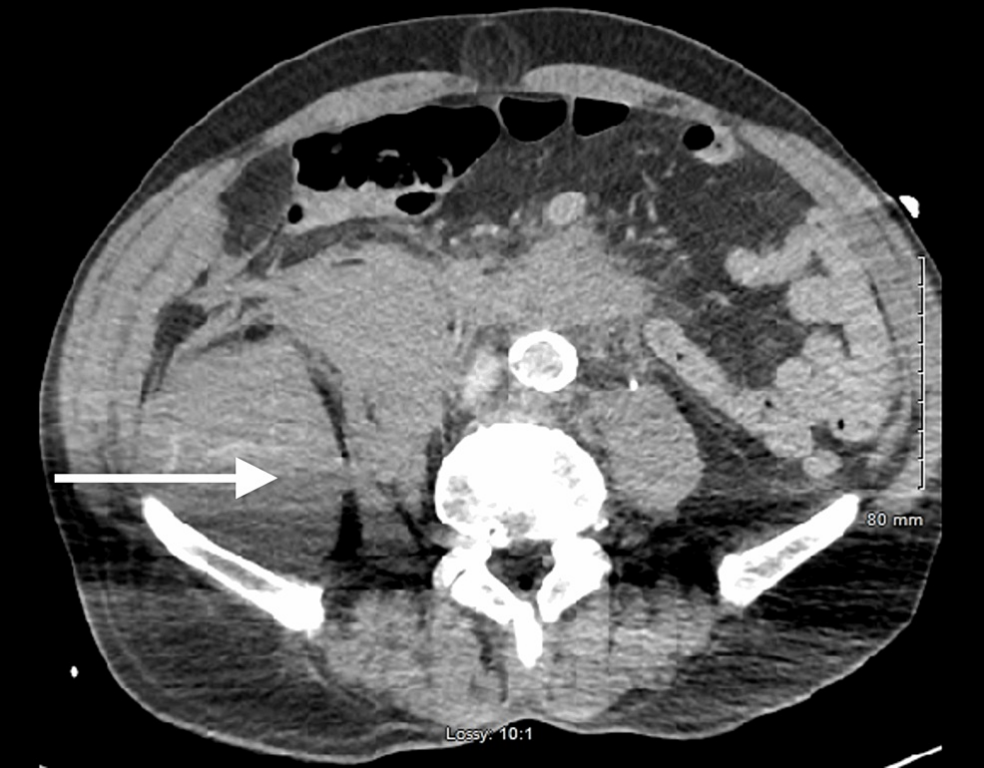
CTA abdomen and pelvis on admission The arrow shows a right iliacus hematoma with contrast extravasation.

A 79-year-old male with a history of glaucoma presented to Desert Regional Medical Level I Trauma Center as a transfer for higher level of care after a fall from a bicycle. The accident took place approximately two weeks prior to arrival at the hospital. On the day of presentation, the patient had awoken with new onset of right lower quadrant abdominal pain. On examination, initial vital signs were 36.8°C, 114 beats/minute, 17 breaths/minute, a blood pressure of 154/99 mmHg, and 100% saturation on room air. Workup revealed a large right retroperitoneal hematoma with active extravasation prompting a trauma evaluation. Initial laboratory results revealed a white blood cell count of 30,200 cells/µL, hemoglobin level of 8.8 g/dL, hematocrit of 26.2%, platelets of 289 × 10^9^/L, sodium of 135 mEq/L, potassium level of 4.6 mEq/L, carbon dioxide level of 20 mmHg, blood urea nitrogen of 21 mg/dL, creatinine of 1.5 mg/dL, glucose of 142 mg/dL, and lactic acid of 4.1 mg/dL. A CT angiography (CTA) of the abdomen and pelvis was performed upon arrival to the emergency department. Results of the CTA were reported as: An iso-dense iliacus hematoma with scattered patchy blush of contrast was seen only at the five-minute delayed venous phase compatible with acute hemorrhage. The hematoma measured approximately 7.6 x 6.8 x 9.4 cm. A small amount of retroperitoneal blood products was seen surrounding the second and third portion of the duodenal C-loop. There was an iso-dense right anterior psoas hematoma measuring 4.1 x 3.1 x 15.3 cm with a small amount of right upper and left upper quadrant hematoma (Figure 1). 

The patient was admitted to the intensive care unit for hemodynamic monitoring and serial hemoglobin and hematocrit levels. During the first night, the patient’s hemoglobin dropped from 8.8 to 6.4 g/dL. Due to concern of ongoing bleeding, Interventional Radiology was consulted and the patient went for retroperitoneal angiography with embolization. During the procedure, gelfoam was used to embolize the anterior division of the right internal iliac artery as well as the right L2 and L3 lumbar arteries with coil embolization of the iliolumbar branch of the right internal iliac artery.

On hospital day 5, the patient continued to show signs of bleeding and a repeat CT was performed showing a new pseudo-aneurysm adjacent to the right common femoral artery without any signs of active bleeding. This time, it was decided to take the patient to the operating room for an exploratory laparotomy and a retroperitoneal exploration with packing for hemorrhage. On hospital day 8, he was returned to the operating room for re-exploration, removal of retroperitoneal packing, washout, and closure of the abdomen. On hospital day 10, he continued to show signs of active bleeding and a repeat CT abdomen and pelvis showed an increase in size of the retroperitoneal hematoma. A Code Blue was called for pulseless electrical activity (PEA), which he responded to well after a single ampule of epinephrine. He was returned to the operating room for re-exploration, evacuation of hematoma, and retroperitoneal packing. The patient remained stable. On hospital day 13, he was returned to the operating room for an additional re-exploration, ligation of a bleeding vessel, and abdominal closure. A drain was placed and he had continued drainage of blood from the retroperitoneum. Over his hospital course, the patient required daily blood transfusions to maintain his hemodynamic stability. In total, the patient was administered 53 total units, which included 30 packed red blood cells, 14 plasma, five platelets and four cryoprecipitate transfusions.

On hospital day 16, a hematology consultation was requested. The patient denied having any previous history of bleeding disorders. As advised by hematology, coagulation studies were ordered, including a Bethesda unit assay, a Von Willebrand antigen test, and a FVIII activity level. While the workup was pending, the patient received blood transfusions and FEIBA (anti-inhibitor coagulant complex) 100 units/kg every 12 hours. A reflex study for FVIII activity demonstrated low results of 2% (normal range is between 50% to 150%). The FVIII inhibitor Bethesda titer was elevated at 24.4 BU/mL (normal range is below 5 BU/mL). Together, these results confirmed the presence of a FVIII inhibitor. The patient’s FEIBA was continued. He was then started on cyclophosphamide 100 mg PO daily, prednisone 50 mg PO daily, and desmopressin. Upon initiation of these treatments, his bleeding decreased. He remained stable, alert, and oriented. On hospital day 31, the patient was discharged to the Medical Surgery unit and later sent home on hospice and comfort care. A few weeks later, he subsequently passed away in his home.

## Discussion

AHA is a rare hemorrhagic condition that is caused by the presence of inhibitors of FVIII. It often occurs in elderly patients above the age of 65 years old, and in the setting of trauma, autoimmune conditions, or malignancy [1-5]. AHA has also been seen in pregnant women, specifically those that are postpartum. There is a high mortality and morbidity risk in this condition, due to a delay in diagnosis and subsequent treatment [6].

AHA is caused by the development of autoantibody inhibitors to FVIII, an important component of the blood coagulation pathway [1-3]. In AHA, the autoantibody inhibitors act by increasing factor clearance or by inhibiting the factor’s coagulation activity [2]. The coagulation cascade is a sequential activation of clotting factors and co-factors to promote the formation of thrombin. During this cascade, FVIII serves as a co-factor to proteolytic enzyme factor IXa, which subsequently activates factor X. An additional role of FVIII during the coagulation pathway is to regulate Von Willebrand factor (VWF). FVIII stabilizes VWF multimers, making these compounds more subject to degradation by the proteolytic enzyme ADAMTS-13 [4]. The use of FVIII protein is the standard treatment for hemophilia A. This protein serves as a replacement coagulation factor for the treatment of and prophylaxis for bleeding episodes.

Patients with acquired hemophilia present with acute bleeding symptoms without a past medical history of bleeding or coagulation disorders. In majority of cases, laboratory results show a normal prothrombin time (PT), prolonged activated partial thromboplastin time (aPTT), reduced FVIII activity, and the presence of inhibitors detected by the Bethesda assay [1]. Subcutaneous bleeding is most common, as seen in 80% of AHA cases. Other less common forms of bleeding seen in AHA include muscle, gastrointestinal, genitourinary, and retroperitoneal bleeding. In congenital hemophilia, hemarthrosis is a hallmark symptom of the condition. Conversely, acquired hemophilia rarely presents with hemarthrosis [1,6].

Current therapeutic management of AHA involves hemostatic control and immunosuppression [1-3]. In our clinical case, the patient continued to show signs of active bleeding even after multiple blood transfusions were administered. Due to significant levels of FVIII inhibitors in the patient, we provided hemostatic control through the anti-inhibitor coagulant complex, FEIBA, which promotes thrombin formation and blood coagulation. Anti-fibrinolytic agents, such as aminocaproic acid, can also be used to reduce severe bleeding episodes [3]. Additionally, immunosuppressive therapy used for AHA includes corticosteroids, alkylating agents such as cyclophosphamide, or monoclonal antibody medications such as rituximab. These immunosuppressants are used to downregulate the production of clotting factor inhibitors and to promote survival. Due to the risk of recurrence of hemorrhagic events, patients should be closely monitored until complete remission and for several months afterward [1-3]. 

## Conclusions

AHA is a rare condition that has a high risk for morbidity and mortality due to an initial delay in diagnosis and subsequent treatment. High suspicion of AHA should be maintained in patients experiencing severe bleeds without a clear etiology, specifically in the setting of trauma, malignancy, pregnancy, or previous autoimmune conditions. Once a diagnosis of AHA is made, therapeutic management through hemostatic control and immunosuppression should be administered promptly in order to reduce coagulation inhibition and control bleeding.
